# Making America great again requires acting on scientific knowledge

**DOI:** 10.1371/journal.pbio.2004337

**Published:** 2018-02-05

**Authors:** Elizabeth A. Hadly

**Affiliations:** Department of Biology, Stanford University, Stanford, California, United States of America

This Perspective is part of the *Conservation Stories from the Front Lines Collection*

When I was 15 years old, I learned that chlorofluorocarbons (CFCs) from sprays such as shaving cream to pesticides could lead to “…the possibility of end to life on earth” [1]. After I read that article, the world seemed smaller and humanity larger.

The article, published in *The New Yorker*, reviewed a science-to-public-to-politics story about CFCs and their purported impact on Earth’s ozone layer. The article recounted the rapid proliferation of aerosol products during the postwar period, from 4.5 million cans in 1947 to 2.3 billion in 1968. The postwar economy of convenience fueled the rapid scale-up, and by 1970, Brodeur reported, “Practically every product that was conceivably sprayable either had been packaged or was being considered for packaging in aerosol form.”

When I read that article, I was in high school in Vicenza, Italy, where my dad was serving in the military. *The New Yorker* was among the few English-language publications we could easily find. At 15, I knew I was younger than their targeted audience, but I devoured the nonfiction, especially the science. Brodeur’s article, in particular, gripped me.

He explained how aerosol cans and the CFC gases they used as propellants were everywhere, their use doubling annually. And everyone had considered CFCs harmless because they were chemically inert. Everyone, that is, except for one Professor F. Sherwood Rowland. The ubiquity of these compounds in the environment, and even the troposphere, made Rowland, an expert in the chemistry of radioactive isotopes, wonder about their fate.

Rowland would join forces with Dr. Mario Molina, then a chemist in Rowland’s lab at the University of California, Irvine. They had conducted a set of chemical calculations once, then again, and published their findings in *Nature* [2]. They’d discovered that chlorine atoms, when mixed with ozone, would produce runaway chemical reactions that could destroy the ozone layer of the atmosphere—the layer that shields living beings from harmful ultraviolet (UV) radiation. More surprisingly, they found that this destruction of the ozone layer would happen rapidly and additively, influenced by the propellants in the convenient aerosol cans that filled the cupboards and garages of consumers across the nation. By 1975, a year after their findings had been published, over 5 billion such cans were being produced annually—mostly by the United States and Russia—and products from pesticides to personal hygiene to food were streaming out of the cans thanks to these CFCs. Their calculations portended atmospheric disaster.

Not believing that spray cans could do so much damage, Rowland and Molina turned to other experts to check their work, thinking they must have made a mathematical error. They hadn’t. Instead, impending harm to the ozone layer was confirmed by others, including Paul Crutzen [3]. Their figures showed that the dangers of increased solar radiation would lead to an increase of 40,000 skin cancer cases in the US by 2050 (real dangers would turn out to be significantly higher [4]), but more significantly, their calculations intimated that stratospheric temperatures might be altered enough to cause changes in global weather patterns. A National Academy of Sciences special panel then recommended an immediate halt to the purchase of aerosol sprays. The panel recommendations were used by the Natural Resources Defense Council to petition the Consumer Product Safety Commission to demand a ban on CFC-propelled sprays. But, not surprisingly, the businesses producing and using CFCs were not idle. As *The New Yorker* article revealed, they attempted to counter the scientists’ discoveries by suggesting that their conclusions were not real—that CFCs were not truly threatening the ozone layer. However, in those days, our government demanded that the burden of proof be on the CFC-using businesses to show that their product was safe, not the scientists to show that it wasn’t [4, 5]. They required that if manufacturers did not demonstrate that CFCs were safe within two years, CFCs would be banned. The Environmental Protection Agency was then tasked with determining what a safe level of CFC production would be, if it existed, and given the chops to enforce it.

In less than a year, the scientific community converged to call out the dangers to the future of our planet caused by spray cans, and our government acted. By 1976, nonessential use of CFCs in aerosol cans was prohibited, and by 1978, cans with CFC propellants were banned in the US [4]. In 1987, the rest of the world agreed: United Nations members signed “The Montreal Protocol on Substances that Deplete the Ozone Layer” [6], which was designed to protect our atmosphere. Rowland, Molina, and Crutzen went on to win the 1995 Nobel Prize in Chemistry [4]. They were planetary heroes.

It would be many years until I realized how important this 1975 article and the scientists behind it were to my intellectual development, and how it made me see the world in a new way. Until then, I had thought of Earth, with wildness thriving far from people, as vast and unexplored—a living planet possessing powerful, poorly understood forces that could wipe humanity’s imprints clean in an instant. But the article, even more than NASA’s lunar program with the voices of astronauts speaking to us Earthlings below, inspired me to zoom out and see our planet as tiny—fragile, even.

I went to college and eventually graduate school, studying a combination of fields intended to satisfy my understanding of human–environment interactions through time (including anthropology, biology, climatology, and geology) and was hired as a professor at Stanford University, where one of my departmental colleagues, Steve Schneider, became a mentor and a good friend. At the same time Rowland and Molina were working on CFCs, Steve had been conducting his own atmospheric calculations about the impacts of greenhouse gases. He would become a science champion who described to the world the dangers of “global warming” [7], served as an advisor to many presidents, and modeled life as a citizen-scientist who worked tirelessly to present his highly complex research in a way the public could understand. It was during a scientific celebration honoring Steve’s 60th birthday that I finally met Sherwood Rowland and was able to tell him what his work had meant to me.

That day, a penny dropped. As I presented my own research, witnessed by these two brilliant change-makers, I realized that Rowland’s global perspective and action, and Steve’s communication skills and energy, combined to influence the trajectory of my career and do so to this day.

My research at that time focused on the paleoecological history of Yellowstone National Park using fossil and modern animals to understand the role that climate plays on their ecology and evolution [8–9]. Although my work was targeted at understanding past climate events such as the Little Ice Age and the Medieval Warm Period (now Medieval Climate Anomaly), I also worked to predict the harbingers of future climatic change. What signs would appear in protected areas? Would they mimic the changes I documented from the fossil record or would they be different? After decades of working in Yellowstone, I thought I could see changes that might be answers. The small ponds that dot the northern part of the park were smaller and drying up earlier than when I first arrived in the park in 1982. Some no longer contained water, and I noticed fewer birds were nesting in the riparian vegetation around the ponds. So, in 2008, my graduate student and I repeated a 1993 study, looking at weather data, amphibian diversity, and geomorphic evidence from 49 ponds [10]. What we found astounded me. As the climate warmed, we documented fewer permanent ponds, and ephemeral ponds that dried up earlier or never appeared at all, leaving fewer suitable habitats for amphibians. As a result, across northern Yellowstone there were significantly fewer populations of all common amphibian species—in some cases, they were reduced by half—and all ponds experienced a reduction in the number of species they harbored. Here—in the world’s first national park, theoretically protected from outside activities—was evidence of the reach of humans that I could observe with my own eyes. Climate change was affecting habitats even in wilderness, supposedly untouched by human hands, and loss of those habitats was impacting the diversity and future of species.

The 1975 *New Yorker* article now reads like a hopeful just-so story, a story in which science worked like it was supposed to. Scientists did their research and presented their results to the public and to our government, which in turn helped to craft evidence-based regulations to protect us and our environment. Industries meanwhile adapted and innovated by developing safer alternatives. Science can, and did, save the planet.

Fast forward to the present. Today planetary emergencies abound—biodiversity loss, climate change, sea-level rise, population increase, migration, resource limitation, and pollution [11]. Even as the impacts of human-caused climate change become clearer and clearer and scarier and scarier, those who govern our country are not responding. In fact, quite the opposite—at the national level, there is active dismantling and discrediting of the science that substantiates environmental problems and solutions. This troubles me because that article I read over 40 years ago still inspires me. I learned that the earth was small and fragile but also that science, when combined with a responsive public and an action-driven government, can drive rapid action to protect this planet.

The irony is hard to miss—the mantra these days is “to make America great again.” But a key feature of those bygone days was listening to the lessons that science taught and acting upon them to make a better planet. Today, it is the trepidation that even though we know, we might not act. And that makes me very worried.
